# A Controllability Investigation of Magnetic Properties for FePt Alloy Nanocomposite Thin Films

**DOI:** 10.3390/nano9010053

**Published:** 2019-01-03

**Authors:** Jian Yu, Tingting Xiao, Xuemin Wang, Xiuwen Zhou, Xinming Wang, Liping Peng, Yan Zhao, Jin Wang, Jie Chen, Hongbu Yin, Weidong Wu

**Affiliations:** 1 Science and Technology on Plasma Physics Laboratory, Research Center of Laser fusion, China Academy of Engineering Physics, Mianyang 621900, China; yujianroy@163.com (J.Y.); tingtingxiao@yeah.net (T.X.); wangxuemin75@sina.com (X.W.); xiuwenzhou@caep.cn (X.Z.); 3965@163.com (X.W.); pengliping2005@126.com (L.P.); zhaoyan267@163.com (Y.Z.); chenjie1067@163.com (J.C.); yhp1214@mail.ustc.edu.cn (H.Y.); 2 State Key Laboratory of Advanced Technology for Materials Synthesis and Processing, Wuhan University of Technology, Wuhan 430070, China; swustwj@163.com; 3 School of Materials Science and Engineering, Southwest University of Science and Technology, Mianyang 621000, China; 4 Collaborative Innovation Center of IFSA (CICIFSA), Shanghai Jiao Tong University, Shanghai 200240, China

**Keywords:** pulse laser deposition, FePt alloy, magnetic phase

## Abstract

An appropriate writing field is very important for magnetic storage application of L1_0_ FePt nanocomposite thin films. However, the applications of pure L1_0_ FePt are limited due to its large coercivity. In this paper, the ratios of L1_0_ and non-L1_0_ phase FePt alloy nanoparticles in FePt/MgO (100) nanocomposite thin films were successfully tuned by pulsed laser deposition method. By adjusting the pulsed laser energy density from 3 to 7 J/cm^2^, the ordering parameter initially increased, and then decreased. The highest ordering parameter of 0.9 was obtained at the pulsed laser energy density of 5 J/cm^2^. At this maximum value, the sample had the least amount of the soft magnetic phase of almost 0%, as analyzed by a magnetic susceptibility study. The saturation magnetization decreased with the increase in the content of soft magnetic phase. Therefore, the magnetic properties of FePt nanocomposite thin films can be controlled, which would be beneficial for the magnetic applications of these thin films.

## 1. Introduction

The face-centered-tetragonal (fct) L1_0_-FePt alloy with large magnetocrystalline anisotropy content was regarded as the most promising material for ultra high density perpendicular magnetic recording [1,2]. However, the high coercivity of FePt greatly exceeds the writing field of available heads, which is limited by the head materials [3]. Thus, it is necessary to find a way to reduce the writing field. Exchange coupling between hard magnetic and soft magnetic phase has been proposed to solve this problem. In order to realize the exchange coupling in materials, two or more phases are necessary in the composite [4,5,6]. 

Furthermore, Fe-rich Fe_3_Pt, Pt-rich FePt_3_ or even disordered face-centered cubic (fcc) alloys can exhibit valuable magnetic properties [7,8,9]. Therefore, combing these materials with L1_0_ FePt has attracted the attention of researchers in the past few years [10,11,12,13]. Sun’s group [11] used the reduction of platinum acetylacetonate and decomposition of iron pentacarbonyl in the presence of oleic acid and oleyl amine stabilizers to synthesize FePt alloy with different Pt concentrations, and studied the relationship between coercivity and Pt concentration. Lin [12] fabricated nanocomposite FePt-FePt_3_films by annealing the (Pt/Fe)_10_ multilayer film, and focused on the influence of annealing temperature. Suber [13] systemically studied interactions between hard and soft magnetic phases by thermal treatment of core-shell FePt (Ag)@Fe_3_O_4_. However, most of the methods to prepare FePt exchange coupling materials involve chemical synthesis, which is expensive and cannot precisely control the proportion of the components. Moreover, the annealing process, which is required for the formation of L1_0_ phase, will lead to undesired particle agglomeration, giving rise to clusters of individual particles [14,15,16]. Since pulse laser deposition (PLD) has the advantages of fast growth rate and easily adjustable process parameters, the prepared samples are free of impurities [17]. Therefore, PLD can be developed as a way to fabricate FePt alloy with different component proportions. Furthermore, compared with other chemical synthesis methods, it is easy to embed FePt nanoparticles in a matrix by PLD method, which can effectively prevent the agglomeration of FePt particles [18]. 

In this work, FePt nanoparticles with different proportions of Pt and Fe were embedded in epitaxial MgO by a PLD method. The ratio of Pt and Fe was confirmed by X-ray photoelectron spectroscopy (XPS). X-ray diffractometer (XRD), and high resolution transmission electron microscope (HRTEM) equipped with an energy dispersive X-ray detector (EDX) were used to analyze the structure of samples with different components. The magnetic properties were measured by superconducting quantum interference device (SQUID). The influence of the pulsed laser energy density of PLD on the structure and magnetic properties of samples was studied. 

## 2. Experimental

FePt/MgO nanocomposite films were grown on MgO (100) substrate by pulsed laser deposition (PLD), which equipped a KrF excimer laser (Anhui Institute of Optics and Fine Mechanics (Hefei, China)) with a wavelength of 248 nm and pulse width of 25 ns. The pure MgO target (purity 99.99%) and an alloy target with a composition of Fe_50_Pt_50_ were used to fabricate the FePt/MgO nanocomposite films under an ultrahigh-vacuum (UHV) system. Before the fabrication of samples, the substrate was heated and degassed for 3 h at 1033 K. The MgO buffer layer was subsequently epitaxially grown on the substrate. Afterwards, the FePt layer of about 6 nm was grown on MgO buffer layer. Subsequently, MgO protective layers were deposited onto the sample. The sample was annealed for about 20 min after the deposition of every MgO layer. The pulsed laser energy density for the growth of MgO was 4 J/cm^2^. For fabricating FePt, the laser energy density was varied from 3, 4, 5, 6 and 7 J/cm^2^, and the samples were labelled as samples 1^#^, 2^#^, 3^#^, 4^#^ and 5^#^, respectively. Finally, all of the as-deposited FePt/MgO nanocomposite films were post-annealed at 1173 K for 4 h under a Ar + H_2_ (5%) flowing gas atmosphere. The experimental parameters for fabricating the FePt/MgO nanocomposite films are listed in Table 1.

XPS analysis (Mg Kα, 1253.6 eV, ThermoFisher Scientific (Waltham, MA, USA)) and EDX (JEOL, Tokyo, Japan) was employed to determine the chemical composition and the proportions of Fe and Pt, and the XPS spectra were fitted using the XPSPEAK41 program and Shirley-type background. The phase composition of the films were identified using XRD (*θ*–2*θ* (symmetric reflection) diffraction geometry, Rigaku, Tokyo, Japan) with CuKα radiation of 1.5418 Å wavelength and HRTEM (JEOL, Tokyo, Japan). The magnetic properties at room temperature were measured by a SQUID (Quantum Design (SanDiego, CA, USA)) in the range of −5 to 5 T.

## 3. Results and Discussion

**Figure 1:**
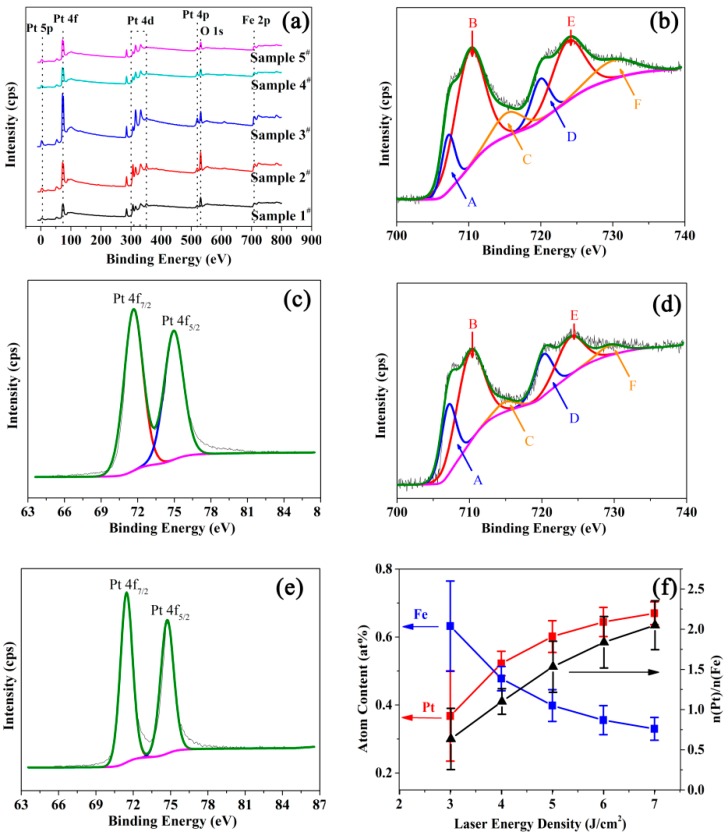
(**a**) XPS survey spectrum for FePt/MgO nanocomposite film, XPS spectrum for Fe 2p (**b**,**d**) and Pt 4f (**c**,**e**) for sample 1^#^ and sample 5^#^, respectively. (**f**) atom content for Fe and Pt, and the Pt/Fe atom ratio for different samples calculated by XPS.

XPS measurement was used to confirm the chemical composition and Pt/Fe ratios for the different samples. It can be seen from the XPS survey spectra of the samples (Figure 1a) that the elements iron, platinum, magnesium and oxygen were present in all the samples. There was no impurity element in FePt/MgO nanocomposite films. Thus, the stability of structure and properties of the samples were ensured. As shown in Figure 1b,d, six peaks are required to fit Fe 2p spectra of sample 1^#^ and sample 5^#^, respectively. The peak A and D near 707.2 ± 0.2 eV and 720.4 ± 0.2 eV correspond to 2p_3/2_ and 2p_1/2_ of pure Fe, respectively. Our previous work has confirmed that there is a peak shift toward to higher binding energy due to the bonding of the Fe and Pt in the single unit [19]. Therefore, the peak B and E located at 710.1 ± 0.2 eV and 724.0±0.2 eV correspond to 2p_3/2_ and 2p_1/2_ of pure Fe of ordered FePt alloy, respectively. And it can be confirm that the Peak A and D are contributed by the Fe of disordered FePt alloy. Besides, there are two small peaks C and F in the detailed spectra of Fe 2p with a binding energy at 714.9 ± 0.2 eV and 729.8 ± 0.2 eV, respectively. They are assigned to Fe in Fe_3_O_4_. This may be caused by the diffusion of oxygen from MgO onto the surface of FePt nanoparticles. The Pt 4f peaks located at 71.50 ± 0.2 and 74.80 ± 0.2 eV corresponding to the Pt 4f_7/2_ and Pt 4f_5/2_ can be identified in Figure 1c,e, respectively. These results confirm that the valence states of Fe and Pt were not affected by the pulsed laser energy density. 

To investigate the molar composition of FePt/MgO composite film, the equations follows was used to calculate the ratio of Fe to Pt with the areas under peaks of elements in the spectra [20].

(1) $$\frac{n_{Fe}}{n_{P}{}_{t}}=\frac{S_{Fe}/g_{Fe}}{S_{P}{}_{t}/g_{P}{}_{t}}$$

where *S* is the area under the peak and g represents the atomic sensitivity factor. The *g* value was set as 10.54 and 1.54 for Fe and Pt, respectively. Figure 1f plots the results as a function of pulsed laser energy density. The ratio of Fe to Pt for FePt/MgO nanocomposite films was calculated with the areas under peaks of elements in the spectra. The Fe atom percentages for sample 1^#^ to sample 5^#^ were 67.46, 48.34, 42.38, 40.50 and 36.56 at%, respectively. Moreover, the Pt/Fe atom ratios for the five samples were calculated as 0.48, 1.07, 1.36, 1.47 and 1.73, respectively. It can be concluded that the ratio of Pt to Fe increased with the increase in the pulsed laser energy density. The proportion of components could be controlled between Fe_67_Pt_33_ and Fe_37_Pt_63._ This phenomenon can be explained as follows. As iron and platinum have different melting points, the element contents evaporated from the target surface were different during the interaction between laser and target. The plasma plume generated by the laser ablation target thereby had different element contents. Therefore, the ratio of Pt to Fe varied with different pulsed laser energy densities.

**Figure 2:**
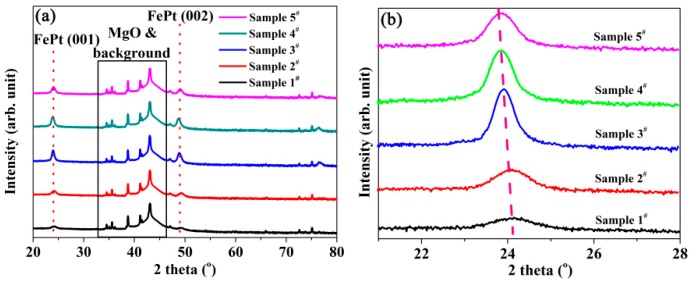
(**a**) The XRD patterns of FePt/MgO nanocomposite films; (**b**) an expanded view of peak (001) of FePt.

In order to confirm the structure of the FePt layer, XRD measurement were performed, and the results are shown in Figure 2. The peaks were labelled using lattice parameters for the bulk tetragonal FePt (a = 0.3847nm, c = 0.3715 nm, P4/mmm, see PDF#43-1359).It can be seen from Figure 2a that the superlattice diffraction peaks (001) and (002) can be distinguished in the patterns for all samples, which indicates that the FePt in all samples was arranged in an ordered tetragonal L1_0_ phase, and that the films had high single-orientation of c-axis. It can be clearly seen from the expanded view in Figure 2b that with the increase in the pulsed laser energy density, the characteristic diffraction peak (001) of FePt shifted to lower angle. According to the Bragg’s formula

(2) 2dsinθ=nλ

where the *d*, *θ* and *λ* are the interplanar crystal spacing, angle between the incident beam and the crystal face and X-ray wavelength, respectively. The shifting to the lower angle indicates the increase in the lattice parameter. This can be explained by Vegard’s law

(3) $$a_{A_{\left(1-x\right)}B_{x}}=(1-x)a_{A}+xa_{B}$$

where a_A(1-x)Bx_ is the lattice parameter of the solution, a_A_ and a_B_ are the lattice parameters of the molar fraction of B in the solution. It was found from the XPS analysis that the Pt/Fe ratio increased with the raise of the pulsed laser energy density, and the lattice parameter for Pt (0.39242 nm) was larger than that of Fe (0.28664 nm). The Pt atom occupied the Fe atom position when the Pt/Fe ratio increased. Therefore, the lattice parameter increases, and the characteristic diffraction peak (001) of FePt shifted to a lower angle for samples with the increase in the pulsed laser energy density.

**Figure 3:**
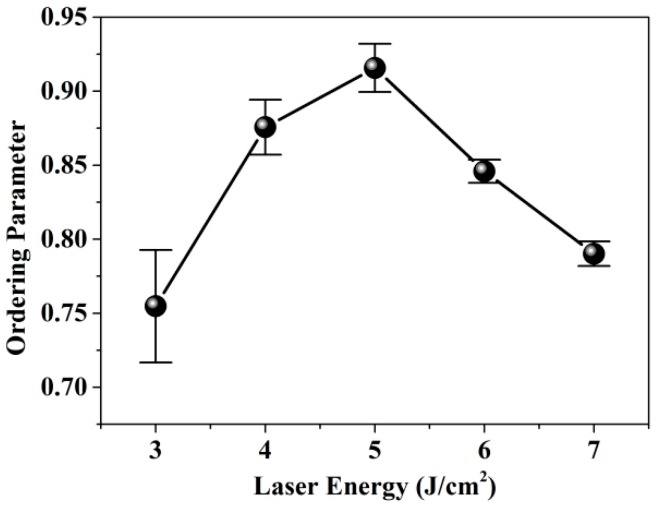
The ordering parameter and FWHM of FePt (001) peak.

To further investigate the growth degree of L1_0_-FePt, the ordering parameter was evaluated as shown below [21]:

(4) $$S={\left[(\frac{I_{\left(001\right)}}{I_{\left(002\right)}})\times {\left(\frac{F_{f}}{F_{s}}\right)}^{2}\frac{{\left(L\times A\times D\right)}_{f}}{{\left(L\times A\times D\right)}_{s}}\right]}^{1/2}=k\times {\left(\frac{I_{\left(001\right)}}{I_{\left(002\right)}}\right)}^{1/2}$$

where *I*_(001)_ and *I*_(002)_ are the peak intensities of FePt (001) and FePt (002); *F*, *L*, *A*, and *D* refer to the structure factor, Lorentz polarization factor, absorption factor, and temperature factor, respectively. The terms f and s represent the fundamental peak and superlattice peak, respectively. The k value (0.59) is obtained by reference [22]. Figure 3 presents the plot of ordering parameter S as a function of pulsed laser energy density. With the increase in the laser energy density, the ordering parameter increased. When the laser energy density was 5 J/cm^2^ for sample 3^#^, and the ordering parameter reached a maximum of 0.90 and then decreased. The reason for this phenomenon can be explained as follows. The excess Pt over the stoichiometric composition could help with atomic diffusion while too much content would affect the structure of L1_0_ order phase [23].

**Figure 4:**
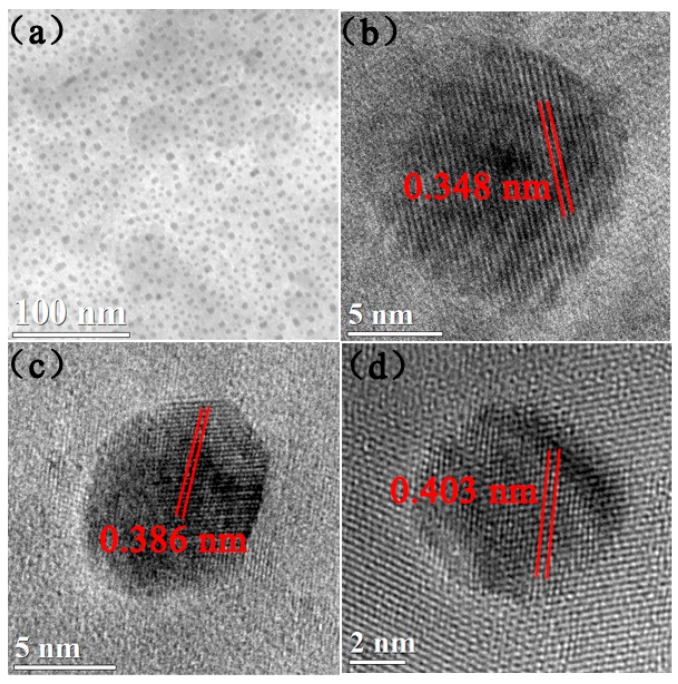
(**a**) The top-view TEM images of the samples: 3^#^; the HRTEM images of samples: (**b**) 1^#^; (**c**) 3^#^ and (**d**) 5^#^.

Figure 4 shows TEM images of the FePt/MgO nanocomposite films. It can be seen from Figure 4a that the FePt nanoparticles were well-separated in the MgO matrix, which indicated that the nucleation regime of FePt in samples was Volmer-Weber-like growth [24]. Figure 4b–d are the HRTEM images of samples 1^#^–3^#^, respectively. It can be seen from the images that changing the pulsed laser energy density did not influence the morphology of FePt/MgO nanocomposite films. The FePt nanoparticles remained spherical and embedded in the MgO matrix. Moreover the superstructure became evident from the alternating bright and dark contrast of the lattice planes. This was due to the largely different electronic scattering cross sections for Fe and Pt [25]. However, with the increase in the pulsed laser energy density, the lattice parameter of the FePt nanoparticles changed. For sample 3^#^ shown in Figure 4c, the lattice parameter was 0.386 nm, which indicates the existence of L1_0_ FePt. And the EDX results for sample 3^#^ (shown in Figure S1 and Table S1) indicate that the Fe/Pt ratio is about 50:50. This result is consistent with the XPS analysis. For samples 1^#^ and 5^#^, as shown in Figure 4b,d, the lattice parameterswere0.348 and 0.403 nm, respectively. It indicates that the lattice parameter of the FePt alloy increased with the increase in pulsed laser energy density. This result was consistent with the XRD analysis.

**Figure 5:**
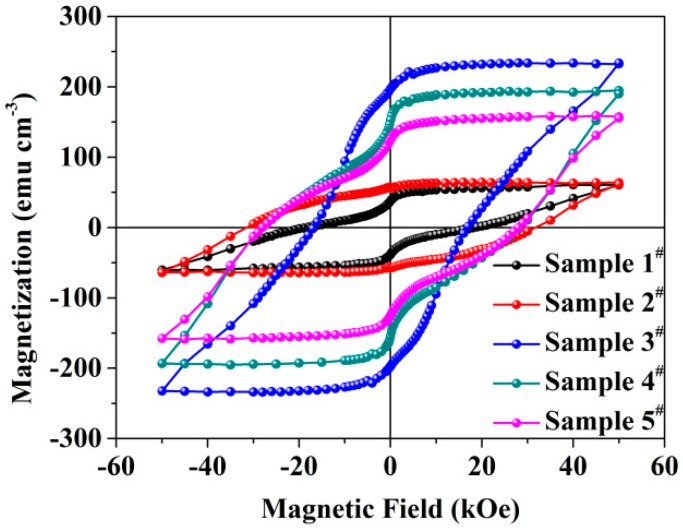
Out-plane field dependence of magnetization recorded at 300K for FePt/MgO nanocomposite films.

Magnetic properties of FePt/MgO nanocomposite films fabricated by different pulsed laser energy densities were measured by SQUID. Figure 5 shows the out-plane hysteresis loops of all samples. As seen from Figure 5, all the samples showed strong ferromagnetic properties. At low magnetic field there was an obvious weak kink when the hysteresis passed through the magnetization axis for samples 1^#^, 4^#^ and 5^#^. However, the hysteresis loops of samples 2^#^ and 3^#^ showed almost smooth curves at low magnetic field. This phenomenon suggests that there was a composite phase consisting of a hard magnetic phase (L1_0_) and soft magnetic phase (A1 or L1_2_) [26]. The reason for the appearance of kinks in the hysteresis curve was possibly the exchange coupling between the hard and soft magnetic phases [27,28]. In general, for mixed magnetic materials, increasing the percentage of soft magnetic phase will enlarge the kink. Therefore the decrease in magnetization at the kink can be used to estimate the fraction of soft magnetic phase in the mixture. Combined with the XPS result, it was evident that the different samples had different Pt/Fe ratios. For the Fe-rich sample 1^#^, the amount of soft phasewas about 47.5%, which may contain FePt fcc phase or Fe_3_Pt, while samples 4^#^ and 5^#^ probably contained FePt fcc phase or FePt_3_ except FePt fct phase because they contained more Pt than Fe. Also, the amount of soft phase for samples 4^#^ and 5^#^ were about 28.1% and 37.6%, respectively. Sample 2^#^ and sample 3^#^, which showed almost smooth curve near the magnetization, had almost 0% decrease at the kink. This conclusion was consistent with the XRD results qualitatively. 

**Figure 6:**
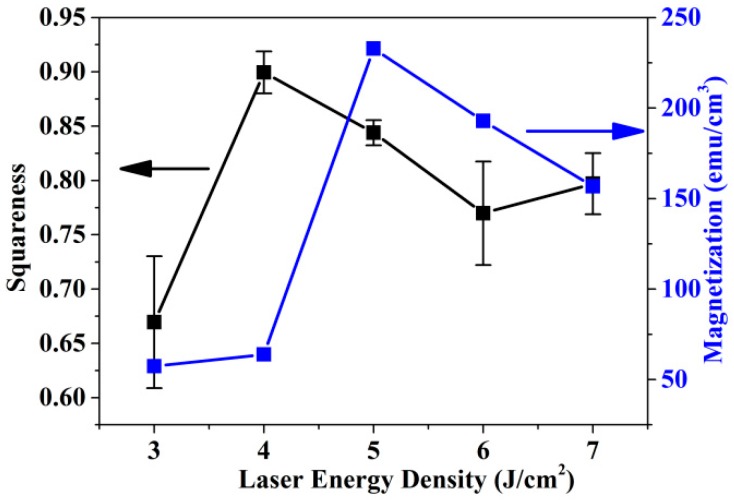
The squareness and saturation magnetization (M_s_) of FePt/MgO nanocomposite films.

Figure 6 shows the squareness and saturation magnetization (M_s_) for samples 1^#^ to 5^#^. The saturation magnetization of sample 3^#^ was 234.1 emu/cm^2^. The saturation magnetization decreased as the pulsed laser energy density changed. For Fe-rich samples 1^#^ and 2^#^, the saturation magnetizations were 60.7 and 64.6 emu/cm^2^, respectively. For samples 4^#^ and 5^#^, which contained more Pt, the saturation magnetizations were 194.8 and 159.1 emu/cm^2^, respectively. The variation trend of the saturation magnetization was consistent with the ordering parameter calculated by XRD patterns. The higher the ordering parameter was, the larger was the saturation magnetization. This can be attributed to the soft magnetic phase which reduces the mean atomic magnetic moment. Therefore the samples with lower ordering parameter had less saturation magnetization [29]. It can also be deduced that the FePt/MgO nanocomposite film with almost equal Fe-Pt ratio had larger saturation magnetization than those with unequal Fe-Pt ratio. Moreover the saturation magnetizations of Pt-rich FePt/MgO nanocomposite films were larger than those of Fe-rich samples. This was because to that a small quantity of Pt-rich sample improved the ordering of FePt [11]. 

The squareness ratio (M_r_/M_s_) of FePt/MgO nanocomposite films is shown in Figure 6. It can be seen that the squareness ratios of samples 2^#^ and 3^#^ were larger than 0.8, and were higher compared to other samples. This demonstrates that the equal Fe-Pt ratio was beneficial with improving the squareness ratio. It is worth noting that sample 2^#^ exhibited large squareness ratio and small saturation magnetization. This was possibly because the Pt content in sample 2^#^ was less than that in sample 3^#^, which made the ordering process incomplete. Adjusting the squareness ratio is helpful for reduction of media noise in magnetic recording media [27]. 

The magnetic properties of FePt/MgO nanocomposite films indicated that the method used in this study was feasible. By adjusting the ratio of Fe and Pt in the nanocomposite films, the saturation magnetization and coercivity can be tuned at high values. Thus the saturation magnetization values can be controlled without sacrificing much coercivity, which can provide advanced magnets for future applications in high density power and date storage [30,31,32].

## 4. Conclusions

In conclusion, PLD was used to fabricate FePt alloy films with different contents of Fe and Pt. The proportion of components was controlled between Fe_67_Pt_33_ to Fe_37_Pt_63_ by adjusting the pulsed laser energy density. When the ratio of Fe and Pt was almost 1, the ordering parameter reached 0.9. Furthermore, with the increase in the content of Fe and Pt, the characteristic diffraction peak (001) of FePt shifted to lower and higher angles, respectively. The films with Fe to Pt ratio of almost showed large squareness ratio, while the slightly Pt-rich film had the largest saturation magnetization. Modifying the magnetic properties by tuning the proportion of components via PLD would be helpful for reduction of media noise in magnetic recording media. 

## Figures and Tables

**Table 1:** Experimental parameters for FePt/MgO nanocomposite film fabrication.

Experiment Conditions	Experiment Parameters
background vacuum	3.0 × 10^−6^ Pa
working vacuum	5.0 × 10^−5^ Pa
target	MgO purity > 99.99% FePt purity > 99.99%
substrate	MgO (100)
laser pulse frequency	2 Hz
number of pulses	MgO: 1200 pulses
distance between the target and substrate	5 cm

## Supplementary Materials

The following are available online at http://www.mdpi.com/2079-4991/9/1/53/s1, Figure S1: The TEM image of sample 3^#^, Table S1: The EDX results of sample 3^#^.
