# Supplementary material for: A Controllability Investigation of Magnetic Properties for FePt Alloy Nanocomposite Thin Films
Source: Nanomaterials (Basel). 2019 Jan 3;9(1):53. doi: 10.3390/nano9010053 (PMC6359714; doi:10.3390/nano9010053)
Supplement: Supplementary file 1 [file nanomaterials-09-00053-s001.pdf]

Supporting Information

A Controllability Investigation of Magnetic Properties for FePt Alloy Nanocomposite Thin Films

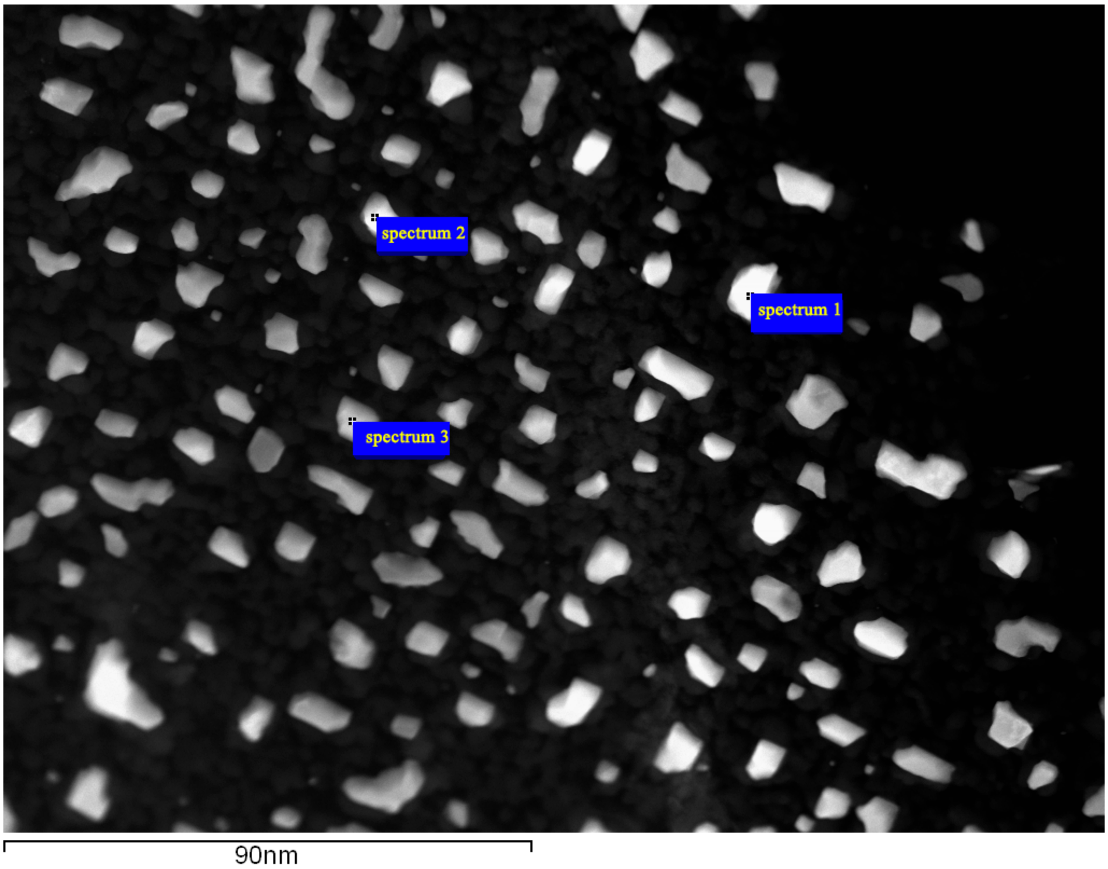

Figure S1. The TEM image of sample 3#.

Table S1. The EDX results of sample 3#.

Spectrum	Fe (wt%)	Pt (wt%)	Fe (Atom %))	Pt (Atom %)
**Spectrum 1**	23.73	76.27	52.09	47.91
**Spectrum 2**	22.84	77.16	50.83	47.91
**Spectrum 3**	21.08	78.92	48.27	51.73
**Average**	22.55	77.45		
**Standard deviations**	1.35	1.35		
**Maximum**	23.73	78.92		
**Minimum**	21.08	76.27		
